# Avelumab as neoadjuvant therapy in patients with urothelial non-metastatic muscle invasive bladder cancer: a multicenter, randomized, non-comparative, phase II study (Oncodistinct 004 - AURA trial)

**DOI:** 10.1186/s12885-021-08990-3

**Published:** 2021-12-02

**Authors:** Nieves Martinez Chanza, Louisa Soukane, Philippe Barthelemy, Aurélien Carnot, Thierry Gil, Vinciane Casert, Vincent Vanhaudenarde, Brieuc Sautois, Lionel Staudacher, Jan Van den Brande, Stephane Culine, Emmanuel Seront, Marco Gizzi, Simone Albisinni, Thibault Tricard, Jean Christophe Fantoni, Marianne Paesmans, Rafael Caparica, Thierry Roumeguere, Ahmad Awada

**Affiliations:** 1 Jules Bordet Institute, Université Libre de Bruxelles, Brussels, Belgium; 2 Hopital Erasme, Université Libre de Bruxelles, Brussels, Belgium; 3 Institut de Cancérologie Strasbourg Europe ICANS, Strasbourg, France; 4 Centre Oscar Lambret, Lille, France; 5 Centre Hospitalier Universitaire de Ambrois Paré, Mons, Belgium; 6 Clinique Sainte-Elisabeth, UCL, Namur, Belgium; 7 University Hospital of Liege (CHU Sart Tilman), Liège, Belgium; 8 Hopital Paris Saint Joseph, Paris, France; 9 University Hospital Antwerp, UZA, Antwerp, Belgium; 10 Hopital Saint-Louis, Paris, France; 11 Hopital de Jolimont, Jolimont, Belgium; 12 Grand Hopital de Charleroi, Charleroi, Belgium

**Keywords:** Avelumab, Bladder cancer, Checkpoint inhibitor, Immunotherapy, Neoadjuvant, PD-1 blockade, Urothelial carcinoma

## Abstract

**Introduction:**

Cisplatin-based neoadjuvant chemotherapy (NAC) followed by surgery is the standard treatment for patients with non-metastatic muscle invasive bladder cancer (MIBC). Unfortunately, many patients are not candidates to receive cisplatin due to renal impairment. Additionally, no predictive biomarkers for pathological complete response (pCR) are currently validated in clinical practice. Studies evaluating immune checkpoint inhibitors in the peri-operative setting are emerging with promising results. Clinical trials are clearly required in the neoadjuvant setting in order to improve therapeutic strategies.

**Methods and analysis:**

Oncodistinct 004 – AURA is an ongoing multicenter phase II randomized trial assessing the efficacy and safety of avelumab single-agent or combined to different NAC regimens in patients with non-metastatic MIBC. Patients are enrolled in two distinct cohorts according to their eligibility to receive cisplatin-based NAC. In the cisplatin eligible cohort, patients are randomized in a 1:1 fashion to receive avelumab combined with cisplatin-gemcitabine or with dose-dense methotrexate-vinblastine-doxorubicin-cisplatin. In the cisplatin ineligible cohort, patients are randomized at a 1:1 ratio to paclitaxel-gemcitabine associated to avelumab or avelumab alone. Primary endpoint is pCR. Secondary endpoints are pathological response and safety.

**Ethics and dissemination:**

The study is approved by ethics committee from all participating centers. All participants provide informed consent prior inclusion to the study. Once completed, results will be published in peer-reviewed journals.

**Trial registration number:**

ClinicalTrials.gov (NCT03674424).

## Strengths and limitations of this study

- This study addresses an important evidence gap regarding the optimal neoadjuvant treatment strategy in patients with a non-metastatic muscle invasive bladder cancer.
- All patients benefit from immune checkpoint inhibitor (avelumab) in the neoadjuvant context, whether they are considered cisplatin eligible or not, and the study evaluates avelumab activity combined with different cytotoxic agents.
- Exploratory translational sub-studies are incorporated in this study in order to move towards an individualized approach driven by biomarkers.
- Lack of long-term follow-up is a limitation for survival outcome evaluation.

## Introduction

The treatment of non-metastatic muscle invasive bladder cancer (MIBC) aims to achieve cure by removing the primary tumor with local treatments and eliminating potential micro-metastasis with systemic therapies. Currently, guidelines recommend cisplatin-based neoadjuvant chemotherapy (NAC) followed by radical cystectomy with bilateral pelvic lymph node dissection as the standard treatment for patients with non-metastatic MIBC [1, 2]. NAC has shown to provide a significant overall survival (OS) benefit when compared to surgery alone; a meta-analysis that included 11 trials and 3005 patients has proved that cisplatin-based NAC yields an absolute 5% benefit in terms of 5-year OS [3].

Despite the survival benefit obtained by NAC, common clinical practice is confronted with unmet needs that must be addressed. First, approximately 40–60% of patients present residual disease despite NAC, which is associated with a higher risk of recurrence [3]. Second, nearly half of the patients diagnosed with non-metastatic MIBC are unfit for cisplatin-based therapy, mostly due to pre-existing comorbidities or low performance status, and currently no alternative options of neoadjuvant treatment exist for these patients [4–6]. Third, there are limited prospective comparisons of NAC approach [7–10]. Last, no validated predictive biomarkers exist to identify which patients benefit from NAC and thus allow treatment individualization.

Immune checkpoint inhibitors (ICI) targeting the programmed death-1 (PD-1) receptor are routinely used in metastatic setting, and their efficacy has generated enthusiasm for their possible utility in early stage disease. The use of the ICI in the neoadjuvant setting aims to generate a potent anti-tumor immune response in the primary tumor providing a huge liberation of neoantigens for cross-priming [11]. Several clinical trials have been developed to assess the role of immunotherapy in the neoadjuvant and peri-operative setting, either combined with chemotherapy or as single agents. Some of these trials have shown promising results with encouraging pathological complete response (pCR) rates so far [12, 13].

The Oncodistinct 004 - AURA trial has been designed to evaluate the efficacy and safety of the ICI avelumab (anti-PDL1) administered as a single-agent or combined with different NAC regimens, in patients with non-metastatic MIBC either eligible or ineligible to cisplatin-based NAC.

## Methods and analysis

### Clinical trial design

AURA is an open-label, interventional, multi-center, randomized, non-comparative phase II study in non-metastatic MIBC patients.

**Fig. 1:**
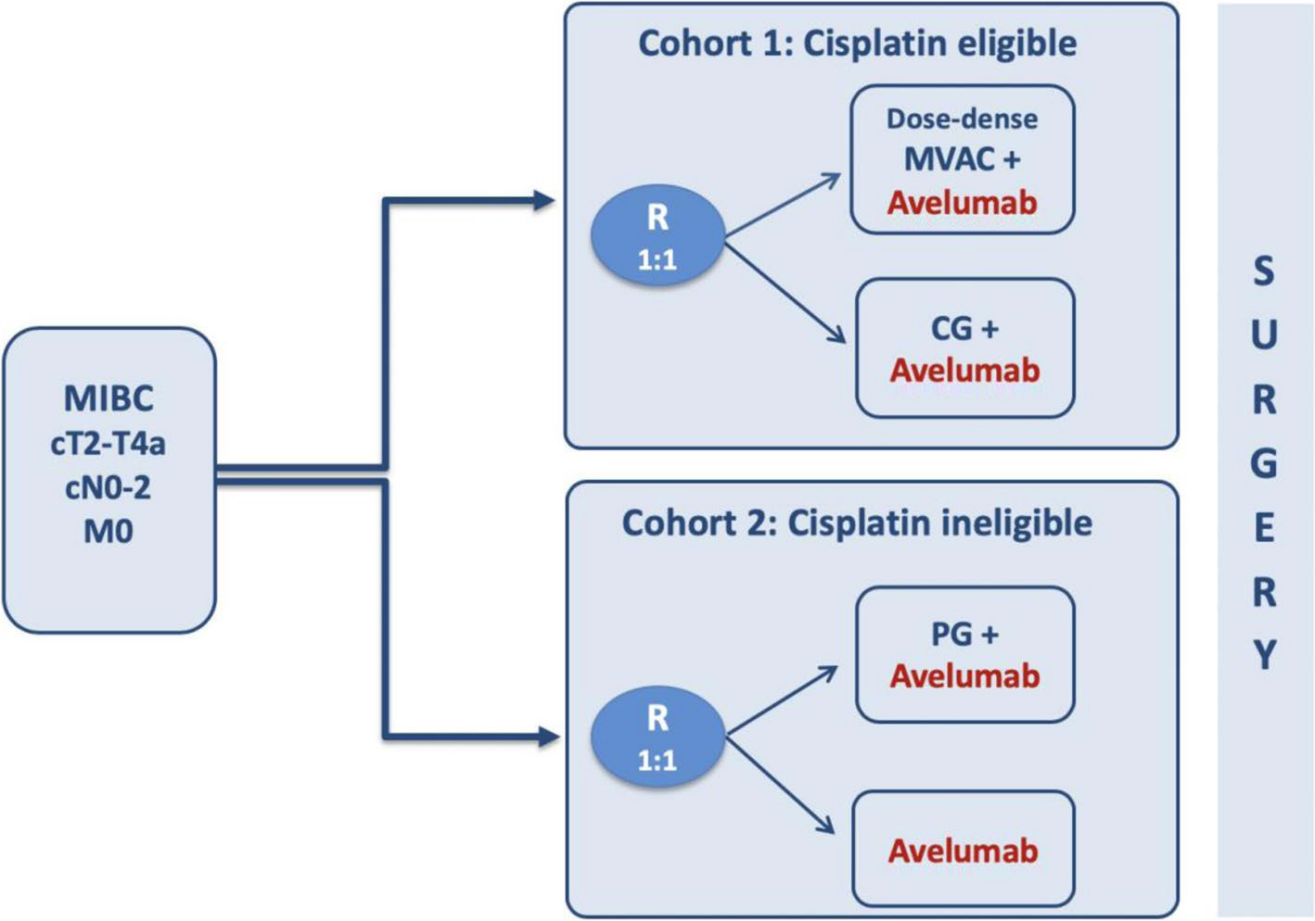
Clinical trial design. Abbreviations – MIBC: Muscle invasive bladder cancer; R: Randomization; MVAC:methotrexate-vinblastine-doxorubicin-cisplatin, CG: cisplatin-gemcitabine; PG: paclitaxelgemcitabine

Patients are divided in two cohorts (cisplatin eligible and cisplatin ineligible) according to their eligibility to receive cisplatin-based NAC [14]. In the cisplatin eligible cohort, patients are randomized in a 1:1 fashion to receive cisplatin-gemcitabine (CG) combined with avelumab or dose-dense methotrexate-vinblastine-doxorubicin-cisplatin (DD-MVAC) in combination with avelumab. In the cisplatin ineligible cohort, patients are randomized at a 1:1 ratio to paclitaxel-gemcitabine (PG) associated to avelumab or avelumab alone (Fig. 1). The randomization procedure is performed using a minimization algorithm with the following stratification factors: institution and clinical lymph node status (N0 vs N+).

### Objectives and endpoints

The main objective of this study is to evaluate the activity of four regimens of neoadjuvant treatment containing avelumab in patients with non-metastatic MIBC. Secondary objective is to assess safety and feasibility of the four neoadjuvant treatment regimens evaluated in the study.

Primary endpoint is pCR, defined as the absence of invasive residual disease (ypT0/Tis) and the absence of microscopic lymph node metastases (ypN0) on the final surgical specimen.

Secondary endpoints include pathologic response, defined as the absence of muscle invasive disease and the absence of microscopic lymph node metastases (<ypT2N0) on the final surgical specimen; and the occurrence of adverse events observed with each regimen. All adverse events will be graded and reported according to the National Cancer Institute Common Terminology Criteria for Adverse Events (NCI-CTCAE version 4.03) from the signing of informed consent form to the end of the study (https://ctep.cancer.gov/protocolDevelopment/electronic_applications/ctc.htm).

### Patient population

Eligible patients must have histologically confirmed urothelial carcinoma or mixed histology with predominant urothelial component (> 50%) and non-metastatic MIBC candidate for surgery as determined by an attending urologist. Tumor stage is based on the standard of care transurethral resection of the bladder tumor sample (T2-T4a). Patients without or with evidence of lymph node disease (N0-N+) are eligible, as long as there is no evidence of distant metastases (M0) on conventional imaging exams (thorax-abdomen-pelvis tomography or thorax tomography and abdomen-pelvis magnetic resonance imaging and bone scan). Performance status 0 or 1 on the Eastern Cooperative Oncology Group (ECOG) and adequate bone marrow and liver function are required. Further details on inclusion and exclusion criteria can be found on clinicaltrials.gov (NCT03674424).

Patients who meet these criteria are enrolled in the cisplatin-eligible or cisplatin-ineligible cohort. Cisplatin-based chemotherapy eligibility includes creatinine clearance ≥60 mL/min (assessed per Cockcroft-Gault formula), peripheral neuropathy ≤ grade 1, hearing impaired ≤ grade 1 and adequate cardiac function (Left Ventricular Ejection Fraction LVEF ≥55%) by MUGA (Multiple-Gated Acquisition) scan or echocardiography [14].

### Procedures

Patients in the cisplatin-eligible cohort receive either CG or DD-MVAC chemotherapy with avelumab. For CG, a dose of gemcitabine 1000 mg/m2 intravenous (IV) on day 1 and day 8 and cisplatin 70 mg/m2 IV on day 1 is administered. Each cycle is given every 3 weeks for a maximum of 4 administrations. For DD-MVAC, methotrexate 30 mg/m2 IV day 1, vinblastine 3 mg/m2 IV day 2, cisplatin 70 mg/m2 IV day 2 and doxorubicin 30 mg/m2 IV day 2 are administered. Each cycle is given every 2 weeks for a maximum of 4 administrations. Avelumab 10 mg/kg IV is given every 2 weeks. Pre-medication and pre-hydration are performed as per manufacturer’s recommendation and local practice. Pegfilgrastim 6 mg subcutaneous is given 24–48 h after DD-MVAC chemotherapy (mandatory), and after CG chemotherapy according to the investigator’s choice.

Patients in the cisplatin-ineligible cohort instead receive either PG with avelumab or avelumab alone. For PG, paclitaxel 80 mg/m2 IV on day 1 and day 15 and gemcitabine 1000 mg/m2 IV on day 1 and day 15 are administered. Each cycle is repeated every 4 weeks for a maximum of 2 administrations. Avelumab 10 mg/kg IV is given every 2 weeks associated to chemotherapy or as single agent. Pre-medication is performed as per manufacturer’s recommendation and local practice.

Adverse events related to chemotherapy should be managed according to local guidelines and dose reductions are recommended if clinically relevant or severe toxicities potentially attributed to chemotherapy occur. If a patient presents severe toxicities (or toxicities that do not resolve with treatment interruption and symptom management) potentially attributed to chemotherapy, treatment with avelumab alone can be continued whereas chemotherapy can be interrupted, according to investigator’s discretion. Dose reductions are not allowed for avelumab. If adverse events potentially attributed to avelumab occur, this medication can be temporarily or permanently interrupted according to investigator’s judgement (https://www.bavencio.com/content/dam/web/healthcare/biopharma/web/Bavencio/BrandSite/nowapproved/USAVE09170349a_BAVENCIO_Dosing_and_Treatment_Guide.pdf).

Radiological re-staging, through the same evaluations performed at baseline, is carried out in the middle of the treatment. On the basis of the investigator decision, the systemic neoadjuvant treatment will be continued or stopped. The European Organization for Research and Treatment of Cancer quality of life questionnaire C30 (EORTC QLQ-C30) is filled by the patients at each treatment cycle (https://qol.eortc.org/questionnaire/eortc-qlq-c30/).

Standard radical cystectomy with bilateral pelvic lymph node dissection is performed 3 to 6 weeks after last administration of neoadjuvant therapy. Surgery is performed by experienced surgeons via an open or robotic assisted approach. The specimen analysis is performed by experienced and dedicated uropathologists.

Patients are assessed for follow-up at 3 months following surgery corresponding at the end of the study period. Additional follow-up visits and staging investigations will be performed as per standard practices at the discretion of the treating physician.

### Statistical analysis

In the cisplatin-eligible cohort, we assumed that a pCR rate of 25% should be reached with NAC alone. We will therefore test, as null hypothesis, that pCR rate with the study treatment regimens, is ≤25%. With a one-sided alpha level of 5%, and a power of 90%, this null hypothesis should be rejected in case the true pCR rate is ≥45%. We will use a two-stage Fleming’s design for each cohort (DDMVAC + avelumab and CG + avelumab). Randomization will be done to allocate patients between DD-MVAC or CG, but the sample size is estimated separately in each of the two arms (no formal between arm comparison planned). Overall, a maximum number of 108 patients needs to be enrolled in order to obtain 98 evaluable patients (49 in each arm). Interim analysis is planned for early efficacy and for futility once 28 evaluable patients have been randomized and followed. A patient is considered evaluable if he/she received at least 1 dose of each medication for his/her corresponding treatment arm and was submitted to surgery with an available pathological specimen for the assessment of pathologic response after neoadjuvant treatment.

In the cisplatin-ineligible cohort, at the time the study was conceived there were no data regarding potential pCR rates expected with ICI alone or combined with NAC in this population. We designed then this part of the trial to be able to demonstrate that achieving pCR is feasible in this population; in other terms, we expect to detect a rate of pCR > 5%. We will therefore test, as null hypothesis, that this rate is ≤5%. With a one-sided test alpha level of 5%, and a power of 90%, this null hypothesis should be rejected in case the true pCR rate is at least 25%. We will use a two-stage Simon design for each of the two cohorts (avelumab alone or with PG). Randomization will be done to choose between those two treatments, but the sample size is estimated separately in each of the two arms (no formal comparison planned), a maximal number of 26 evaluable patients will be required in each arm.

An interim analysis to assess efficacy and safety is planned for each arm. Actual study start was June the 1st 2018 and estimated primary completion date is November the 1st 2022. All data will be prospectively collected. The analysis will be conducted separately for the four groups of patients.

### Biomarker analysis

Exploratory correlative studies are planned (the actual analytical methodologies will only be specified and decided upon at the time these analyses will start). In this study, we will evaluate the immunomodulatory properties of avelumab alone and in combination with different cytotoxic agents and correlate these findings with outcome. In addition, we will investigate the potential mechanisms of sensitivity/resistance to immunotherapy and/or chemotherapy to identify predictive biomarkers that allow the selection of patients who benefit most likely from immunotherapy. Biopsy and surgical tumoral specimens pre- and post- systemic therapy are collected. Blood, urine and stool samples are also collected at baseline, mid-treatment and prior surgery.

### Patient and public involvement

Patients and the public were not involved in the design of this study.

## Discussion

There is level one evidence for cisplatin-based NAC in non-metastatic MIBC patients, and the presence of residual disease is associated with a higher risk of metastatic recurrence rather than locoregional recurrences [1–3]. Achieving a pCR or downstaging to non MIBC on the final surgical specimens with neoadjuvant cisplatin-based combination chemotherapy is associated with high cure rate and increased survival with best outcomes described in patients who attain a pCR defined as ypT0 disease [15–17]. The landmark SWOG 8710 trial demonstrated that three cycles of neoadjuvant MVAC regimen compared to surgery alone improved 5-year OS (57% vs. 43%; *p* = 0.06) [18]. Significant improvement in pCR (38% vs. 15%; *p* < 0.001) and median OS (77 vs. 46 months, *p* = 0.06) were also observed.

The most commonly used cisplatin-based regimens in daily clinical practice are CG and DD-MVAC, although the optimal chemotherapy regimen is still unknown [7, 8, 19, 20]. A retrospective study evaluated 319 patients treated with NAC reporting a higher pCR rate in the DD-MVAC group compared to CG (28.0% vs. 14.6%; *p* = 0.005, respectively) which was correlated with longer survival (7 vs 4.6 years, *p* = 0.001) [9]. Recently, preliminary results on the secondary endpoints of pCR and toxicity from a phase III trial comparing both regimens (CG for 4 cycles and ddMVAC for 6 cycles) were presented [10]. pCR rates favored DD-MVAC over CG regimen (42% vs. 36%; *p* = 0.02, respectively) and grade 3 adverse events were more frequently observed in the DD-MVAC arm including anemia (22% vs. 8%; *p* = 0.00002), febrile neutropenia (7% vs. 2%; *p* = 0.05), nausea/vomiting (10% vs. 3%; *p* = 0.03), and asthenia (14% vs. 4%; *p* = 0.0002). Results related to disease-free survival which is the primary objective are expected for 2021.

There is no evidence today supporting that the administration of non-platinum regimens improves patient outcome in the neoadjuvant setting [1, 2]. Cisplatin ineligibility is common in this population, due to pre-existing comorbidities such as renal dysfunction, hearing impairment, cardiac insufficiency or peripheral neuropathy, or performance status ≥2, or both. As such, 40% of bladder cancer patients are ineligible for NAC [4–6]. Unfortunately, no therapeutic alternative exists today for this population and data suggest that carboplatin is suboptimal [4, 5]. Therefore, there is an urgent need for more effective regimens in the neoadjuvant setting as well as therapeutic alternatives for patients not eligible to cisplatin.

Immunotherapy has demonstrated sustained efficacy with a favorable safety and tolerability profile in advanced urothelial carcinoma patients. Since 2016, five new programmed cell death protein-1/ligand 1 (PD-1/L1) checkpoint inhibitors have been approved for metastatic urothelial carcinoma (atezolizumab, pembrolizumab, avelumab, durvalumab and nivolumab). Consequently, with the significant beneficial impact of immunotherapy in advanced disease, different clinical trials have been developed to evaluate the role of these agents in the neoadjuvant and peri-operative settings aiming to prevent disease recurrence and improve cure rates. The activity of ICI single agent as well as the combination with either ICI, chemotherapy, epigenetic drugs or radiotherapy is being explored in the neoadjuvant setting. The administration of two cycles of atezolizumab (6 weeks treatment) before cystectomy has been evaluated in 95 patients with MIBC in a phase II study (ABACUS). The pCR was 31% (95%CI: 21–41%), with the study achieving its primary efficacy endpoint [12]. Investigators described a correlation between pre-existing activated T-cells and PD-L1 positive tumors with pathological response as well as a therapy resistance with the stromal factors’ expression. Another phase II study (PURE-01), evaluated 3 cycles of neoadjuvant pembrolizumab (6 weeks treatment) in cisplatin eligible and ineligible patients [13]. In a first interim analyses, researchers observed a pCR rate of 42% (95%CI: 28.2–56.8%) among 50 treated patients with a more robust activity in patients whose tumors expressed PD-L1 or presented high mutation burden. Recently, preliminary results of ICI combinations have been presented. Neoadjuvant dual-ICI (ipilimumab+nivolumab) has been tested in the NABUCCO phase 1b study in cisplatin-ineligible patients with stage III urothelial carcinoma, which is in fact a high-risk group, in whom a more intensive approach is justified [21]. Primary endpoint was treatment feasibility with 96% of patients achieving a surgical resection within 12 weeks from first ICI administration. Authors reported a 46% pCR rate and a 58% tumor downstaging. Finally, the BLASST-1 trial evaluated the combination of Cisplatin-Gemcitabine NAC with nivolumab [22]. A pCR of 49% (20/41) and downstaging of 66% (27/41) were achieved. Treatment was well tolerated with immune-related adverse events observed in only 3 patients.

Avelumab (MSB0010718C) is a fully human IgG1 monoclonal antibody that inhibits PD-1/PD-L1 interactions while leaving the PD-1/PD-L2 pathway intact. Unlike other anti– PD-L1/PD-1 antibodies that are approved or in advanced clinical development, avelumab also induces lysis of tumor cells via antibody-dependent cell-mediated cytotoxicity suggesting an additional mechanism of action [23]. Evidence of clinical activity and an acceptable safety profile have been shown in a large, international, multi-cohort, phase I study in patients with refractory advanced solid tumors including advanced urothelial carcinoma [24]. In the dose-escalation part, intravenous infusion of avelumab every 2 weeks was safe and had a predictable pharmacokinetic profile at doses ≤20 mg/kg. The 10 mg/kg dose was selected for study in phase Ib expansion cohorts in a range of tumor types. Results in 249 metastatic urothelial carcinoma patients showed that avelumab is a potential treatment option with a safety profile. In 161 post-platinum patients with at least 6 months of follow-up, the overall response rate was 17% (95% CI 11–24). Grade 3 and higher treatment-related AEs occurred in 8% (21/249), the most common were fatigue (2%), and asthenia, elevated lipase, hypophosphataemia, and pneumonitis (1% each). Recently, avelumab has showed longer survival in first line maintenance in patients whose disease has not progressed with platinum-based induction chemotherapy [25].

The ability to predict response to a specific therapy is still a major challenge in oncology. Currently, no biomarkers are used in the clinical setting to predict response in urothelial carcinoma. However, the evidence for the eventual individualization of treatment in MIBC based on genomics and molecular subtyping is growing. The most important and promising predictive biomarkers to neoadjuvant chemotherapy under investigation are regulators of cell cycle and apoptosis (p53, Bcl-2), pathways involved in DNA repair (BRCA1, ERCC1, ATM, RB, FANCC), gene expression signatures and molecular subtypes. Alterations in DNA damage and repair (DDR) genes have emerged as a biomarker of response to neoadjuvant cisplatin-based chemotherapy in several studies. Loss-of-function mutations in the nucleotide excision repair gene ERCC2 as well as in related DDR genes including ATM, FANCC, and RB1 have been associated with pCR to cisplatin-based chemotherapy and improved survival in MIBC patients receiving cisplatin-based chemotherapy [26–28]. Functional studies have confirmed that many of the clinically identified DDR gene mutations confer loss of DNA repair capacity and drive cisplatin sensitivity in preclinical bladder systems. Predictive biomarkers of response, single or in association, to determine the optimal ICI treatment for patients with bladder cancer are also under evaluation. The most important and promising predictive biomarkers are PD-L1 expression, tumor mutational burden (TMB), immune cell gene expression profiling, CD8+ cells and Granzyme B, and molecular subtyping. PD-L1 expression on surgical specimens has been positively correlated with response in the PURE-01 and NABUCCO trials [13, 21]. In the ABACUS trial a statistically significant correlation between PD-L1 expression levels and response to neoadjuvant ICI was not observed [12]. Tumors with a higher TMB seem more likely to express a high number of neoantigens inducing a more robust response to ICI. In the PURE-01 trial, a non-linear association between higher TMB (scores ≥15 mut/Mb) and pCR was found [13]. The ABACUS trial did not confirm these findings because no correlation was found between high TMB and increased percentage of response to neoadjuvant atezolizumab [12]. It is noteworthy that PD-L1 expression was not correlated with high TMB. In the ABACUS trial, a transcriptional signature of eight genes (IFNG, CXCL9, CD8A, GZMA, GZMB, CXCL10, PRF1, and TBX21), previously described in locally advanced or metastatic urothelial carcinoma, resulted significantly increased in patients responsive to atezolizumab compared to non-responder patients or in patients with disease relapse [12]. Advances in genomic profiling have allowed to classify molecularly the heterogeneous bladder cancer into specific genomic subtypes with similar biomolecular features, prognosis and response to treatment [29]. The luminal-infiltrated subtype, that appears to be resistant to cisplatin-based chemotherapy, has been reported to respond to ICI in patients with metastatic or unresectable bladder cancer. Instead, basal-squamous subtype seems to benefit both from cisplatin-based NAC and from ICI.

Beyond immunotherapy, the antibody-drug conjugates, monoclonal antibodies directed against cancer cell surface proteins, represent a class of emerging therapeutics which have demonstrate clinically meaningful efficacy with relatively favorable toxicity profiles across a range of cancers including advanced urothelial carcinoma. Enfotumab vedotin is a Nectin-4-directed antibody-drug conjugate evaluated in a phase III clinical trial (EV-301) of 608 patients with locally advanced unresectable or metastatic urothelial carcinoma (including those with squamous differentiation or mixed cell types) previously treated with platinum-based chemotherapy and PD-1/PD-L1 inhibitor [30]. Patients were randomly assigned to either enfortumab vedotin or investigator’s choice of chemotherapy (decetaxel, paclitaxel or vinflunine). At median follow-up of approximately 11 months, compared with chemotherapy, enfortumab vedotin improved OS (median 13 versus 9 months, HR 0.70, 95% CI 0.56–0.89), progression free survival (PFS) (median 6 versus 4 months, HR 0.62, 95% CI 0.51–0.75) and overall response rates (41% versus 18%). Sacituzumab govitecan is another antibody-drug conjugate that recognizes Trop-2, a cell-surface glycoprotein highly expressed in most urothelial carcinomas. A recent phase II trial included 113 patients with advanced urothelial carcinoma who experienced prior progression after platinum-based chemotherapy and a checkpoint inhibitor (either with a PD-1 or PD-L1 inhibitor) [31]. The objective response rate was 27% with 5% of compete responses. Median OS and PFS were 5 and 11 months, respectively. Consequently, a randomized phase III trial (NCT04527991) with sacituzumab is currently recruiting patients. Ongoing research will best determine how and when to combine antibody-drug conjugates with anti-PD-1/L1 immunotherapy and/or platinum-based chemotherapy in patients with advanced urothelial cancer.

Given the evidence of clinical activity and manageable safety profile of avelumab in patients with advanced urothelial carcinoma, integration avelumab in the neoadjuvant setting in non-metastatic MIBC patients offers the potential for a new therapeutic approach. Oncodistinct 004 - AURA study assesses efficacy, safety and translational research program of neoadjuvant avelumab single-agent and combined with different cytotoxic agents in cisplatin-eligible and cisplatin-ineligible non-metastatic MIBC patients.

## Conclusions

In the present manuscript, we describe the design of the Oncodistinct 004 - AURA trial testing avelumab alone or combined with different chemotherapy regimens as neoadjuvant strategy in cisplatin-eligible and cisplatin-ineligible non-metastatic MIBC patients. Currently, there are multiple initiatives worldwide aiming to improve the benefits of the current standard-of-care in non-metastatic MIBC. First reports in this field including ICI show feasibility and great potential to significantly improve outcomes. At this time, we encourage participation in clinical trials such as AURA in order to improve outcome and to identify molecular biomarkers for perioperative therapy patient selection.

## Data Availability

Not applicable. No data are presented in this article.

## Abbreviations

CG: Cisplatin-gemcitabine
DD-MVAC: Dose-dense methotrexate-vinblastine-doxorubicin-cisplatin
DDR: DNA damage and repair
ICI: Immune checkpoint inhibitors
IV: Intravenous
MIBC: Muscle invasive bladder cancer
NAC: Neoadjuvant chemotherapy
OS: Overall survival
pCR: Pathological complete response
PD-1: Programmed death-1
PFS: Progression free survival
PG: Paclitaxel-gemcitabine
TMB: Tumor mutational burden
